# A brain-inspired intention prediction model and its applications to humanoid robot

**DOI:** 10.3389/fnins.2022.1009237

**Published:** 2022-10-21

**Authors:** Yuxuan Zhao, Yi Zeng

**Affiliations:** ^1^Research Center for Brain-inspired Intelligence, Institute of Automation, Chinese Academy of Sciences, Beijing, China; ^2^Center for Excellence in Brain Science and Intelligence Technology, Chinese Academy of Sciences, Beijing, China; ^3^National Laboratory of Pattern Recognition, Institute of Automation, Chinese Academy of Sciences, Beijing, China; ^4^School of Artificial Intelligence, University of Chinese Academy of Sciences, Beijing, China

**Keywords:** human-robot interaction, intention prediction, brain-inspired model, spiking neural networks, humanoid robot

## Abstract

With the development of artificial intelligence and robotic technology in recent years, robots are gradually integrated into human daily life. Most of the human-robot interaction technologies currently applied to home service robots are programmed by the manufacturer first, and then instruct the user to trigger the implementation through voice commands or gesture commands. Although these methods are simple and effective, they lack some flexibility, especially when the programming program is contrary to user habits, which will lead to a significant decline in user experience satisfaction. To make that robots can better serve human beings, adaptable, simple, and flexible human-robot interaction technology is essential. Based on the neural mechanism of reinforcement learning, we propose a brain-inspired intention prediction model to enable the robot to perform actions according to the user's intention. With the spike-timing-dependent plasticity (STDP) mechanisms and the simple feedback of right or wrong, the humanoid robot NAO could successfully predict the user's intentions in Human Intention Prediction Experiment and Trajectory Tracking Experiment. Compared with the traditional Q-learning method, the training times required by the proposed model are reduced by (*N*^2^ − *N*)/4, where N is the number of intentions.

## 1. Introduction

The research trend of the new generation of robots is to make robots participate in human life, and improve the naturalness and flexibility of interaction between humans and robots through human-robot interaction technology. Robots that can successfully predict user's intention and take appropriate actions according to the intention can effectively improve interaction efficiency and user experience. Researchers have made significant progress in user intention prediction modeling. These studies use a variety of frameworks or models to enable robots to predict users' intentions in specific human-robot interaction tasks. These frameworks or models use a variety of methods, such as probabilistic graphical models, deep learning techniques, and other methods that include extreme learning machine algorithms, etc.

There are many studies on the application of the probability graph model to human intention prediction. Song et al. (2013) proposes a probabilistic graphical model for predicting human manipulation intention from image sequences of human-object interaction. The model can enable the robot to successfully infer intention in a house-hold task which contains four intentions: hand-over, pouring, tool-use, and dish-washing. Vinanzi et al. (2019) proposes a novel artificial cognitive architecture to predict the intentions of a human partner. The architecture contains unsupervised dynamical clustering of human skeletal data and a hidden semi-Markov chain. With the architecture, the iCub robot can engage in cooperative behavior by performing intention reading based on the partner's physical clues. Besides that, Yu et al. (2021) proposes a Bayesian method for human motion intention in a human-robot collaborative task. Dermy et al. (2017) and Luo and Mai (2019) built models based on Probabilistic Movement Primitives for human intention prediction. Their models are verified in gaze guidance experiment or tabletop manipulation task.

Deep learning techniques, especially deep long short-term memory (LSTM) neural network, have also been used to predict human intentions. Yan et al. (2019) presents an LSTM neural network to recognize human intention. They designed a human-robot collaboration environment using a UR5 robot and a Kinect V2 depth camera. The experimental results show that the 2-layers deep LSTM network enables the robot to understand the human intentions even with only 40% of the motion sequences. Liu et al. (2019) presents a deep learning system combing convolutional neural network (CNN) and LSTM, and this system could accurately predict the motion intention of the human in a desktop disassembly task.

In addition, there are other methods for human intention recognition. Wang et al. (2021) proposes a teaching-learning-prediction (TLP) framework, which enables robots to learn and predict human hand-over intentions in collaborative tasks. The robot learns the human demonstrations *via* the extreme learning machine (ELM) algorithm, which realizes the robot's learning and prediction of human hand-over intentions in collaborative tasks. The experimental results show that the framework can enable the robot to effectively predict the human hand-over intention and complete the hand-over task. Since the framework enables robots to learn through human demonstrations, it can reduce human manual-programming efforts and improve the efficiency of human-robot collaboration. Lin et al. (2017) develops a human intention recognition framework in human-robot collaboration scenarios. The framework contains an inverse-reinforcement learning system to find the optimal reward function of the policy and a Markov-Decision process to model human intention. They use a coffee-making task and a pick-and-place task to verify the validity of the model and obtained the desired results. Li et al. (2020) proposes a task-based framework to enable robots to understand human intention from natural language dialogues. The framework includes a language semantics module for extracting instruction keywords, a visual object recognition module for identifying objects, and a similarity computation module for inferring intention based on the given task. With this framework, the robot could comprehend human intentions using visual semantics information.

It can be seen that most of the current studies use relatively complex methods to complete specific human-robot interaction tasks, and few studies use brain-inspired cognitive computational modeling methods to solve intention prediction tasks. Brain-inspired cognitive computational modeling is a method that draws on the results of neuroimaging studies on cognitive tasks, proposes feasible neural pathways and network structures, and conducts modeling based on the spiking neuron model.

Here, based on the neuroimaging studies of reinforcement learning, we propose a brain-inspired intention prediction model to enable the robot to perform actions according to the user's intention. Based on the brain-inspired network structure, the humanoid robot NAO could successfully predict the user's intentions in Human Intention Prediction Experiment and Trajectory Tracking Experiment only by using the spike-timing-dependent plasticity (STDP) mechanisms and the simple feedback of right or wrong.

## 2. Materials and methods

### 2.1. Architecture of the brain-inspired intention prediction model

**Figure 1:**
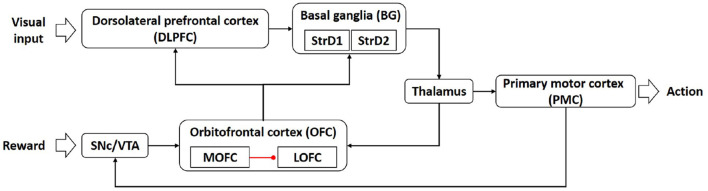
The architecture of the brain-inspired intention prediction model.

The architecture of the brain-inspired intention prediction model is shown in Figure 1.

The dorsolateral prefrontal cortex (DLPFC) is responsible for representing state information (Barbey et al., 2013). In our computational model, the DLPFC receives input from visual cortex and abstractly represents the visual information.

Popular theories implicate that the basal ganglia (BG) are responsible for action selection (Stocco et al., 2010; Friend and Kravitz, 2014). The striatum D1 (StrD1) and striatum D2 (StrD2) are components of BG (Villagrasa et al., 2018). In our computational model, the BG is used for intention prediction, that is, BG selects the actions that conform to the user's intention according to the visual information represented by DLPFC.

The thalamus is generally considered to be a relay station, transmitting information between different cerebral cortex (Hwang et al., 2017). In our computational model, the thalamus acts as a relay station to transmit information from BG to PMC and OFC.

The primary motor cortex (PMC) is a critical area for controlling the execution of movement (Kakei et al., 1999). In our computational model, the PMC is used to control the actions of the robot.

The substantia nigra pars compacta and ventral tegmental area (SNc/VTA) play important roles in reward cognition (Haber and Knutson, 2010; Zhao et al., 2018). In our computational model, the SNc/VTA receives the user's feedback and determines the pathway of information transmission. When the feedback information is positive, SNc/VTA combines the information from PMC and transmits the stimulation to OFC_2 (a sub-region in orbitofrontal cortex). When the feedback information is negative, the stimulation of SNc/VTA is 0.

The orbitofrontal cortex (OFC) is considered as a critical frontal region for memory formation (Frey and Petrides, 2002). The sub-region medial orbitofrontal cortex (MOFC) and lateral orbitofrontal cortex (LOFC) respond to positive reward (O'Doherty et al., 2001) and negative reward (Kringelbach, 2005). In our computational model, the OFC contains OFC_1 and OFC_2, MOFC and LOFC. The OFC_1 and OFC_2 are used to receive and store information from the thalamus and SNc/VTA. When the feedback information is positive, the MOFC receives stimulation from OFC_1, and the LOFC receives stimulation from OFC_2 and is inhibited by MOFC at the same time. When the feedback information is negative, only the LOFC receives the stimulus from OFC_1 and OFC_2. Then the MOFC transmits the information to DLPFC and StrD1 in BG, and the LOFC transmits the information to DLPFC and StrD2 in BG.

### 2.2. Model implementation

**Figure 2:**
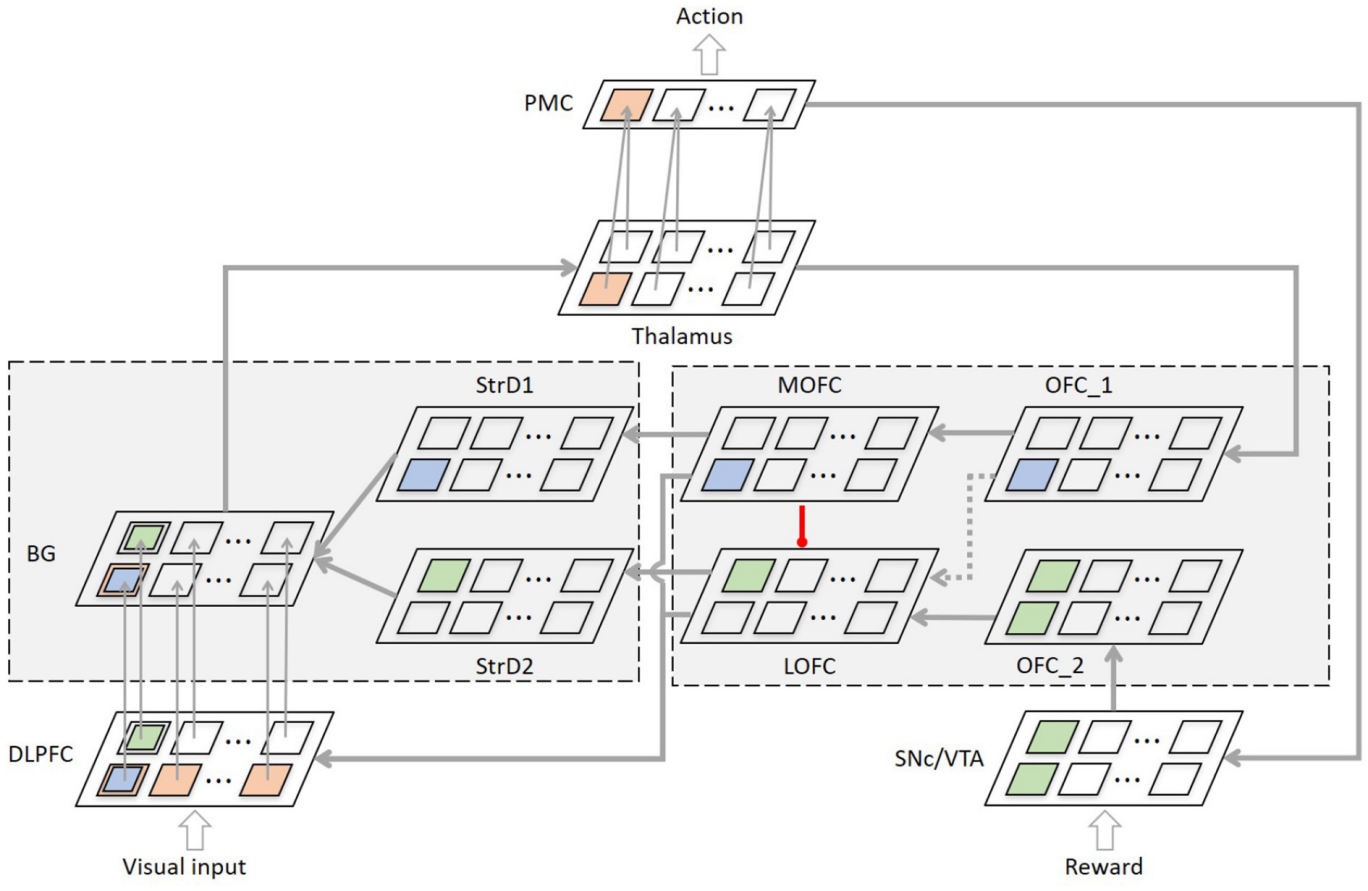
The concrete neural network architecture of the brain-inspired intention prediction model. The sizes of the regions depend on the number of intentions. Taking the number of intentions and actions as 12, the size of different regions except PMC in the model is 12 x 12, and the size of PMC is 1 x 12.

The concrete neural network architecture of the model is shown as Figure 2.

In order to describe one training process of the model more directly, in Figure 2, we use orange neurons, blue neurons and green neurons to represent the neurons be activated in one training process. **1. The intention prediction process:** (a) The visual information of image category *1* is input into DLPFC, and all neurons representing this category in DLPFC are activated (orange neurons in DLPFC); (b) After the synaptic weight matrix calculation between DLFPC and BG, the neuron representing intention *1* in BG is activated (orange neuron in BG); (c) Thalamus receives the results of BG (orange neuron in Thalamus) and passes the information to PMC to control motor generation (orange neuron in PMC). **2. The positive reward process**, if the user gives *positive reward* into SNc/VTA: (a) The OFC_1 receives the stimulation form Thalamus (blue neuron in OFC_1). The SNc/VTA combines the information from PMC and transmits the stimulation to OFC_2. And all neurons representing this action are activated (green neurons in OFC_2). (b) Then the stimulation of OFC_1 and OFC_2 are transmitted to MOFC (blue neuron in MOFC) and LOFC respectively, and LOFC is simultaneously inhibited by MOFC (green neurons in LOFC). (c) MOFC transmits the information to BG *via* StrD1 and to DLPFC at the same time. LOFC transmits the information to BG *via* StrD2 and to DLPFC at the same time. (d) The synaptic weight between DLPFC and BG is updated according to the time difference between the neurons firing. ***In short, the connection between image category 1 and*** ***intention 1 is strengthened, and the connections between image category 1 and other*** ***intentions are weakened*. 3. The negative reward process**, if the user gives *negative reward* into SNc/VTA: (a) The OFC_1 receives the stimulation form Thalamus, then transmits the information to LOFC. (b) LOFC transmits the information to BG *via* StrD2 and to DLPFC at the same time. (c) The synaptic weight between DLPFC and BG is updated according to the time difference between the neurons firing. ***In short, the connection between image category 1 and intention 1 weakened*,** ***while the connections between image category 1 and other intentions remained*** ***unchanged*.**

We use the Izhikevich neuron model to build the computational model. The Izhikevich neuron model achieves a good balance in biologically plausible and computational efficiency (Izhikevich, 2003). The neuron model is described as Equations (1) and (2). The variable v represents the membrane potential of the neuron and u represents a membrane recovery variable. And *a*, *b*, *c*, and *d* are dimensionless parameters. If the membrane voltage v is greater than 30 mV, the membrane voltage and the recovery variable are reset according to the Equation (2). I is input, calculated by Equation (3). The *W*_*ij*_ is the synaptic weight between presynaptic neuron and postsynaptic neuron, and the *O*_*ij*_ is the output of presynaptic neuron. If multiple neurons fire at the same time, the neuron with the largest membrane voltage will inhibit other neuronal firings. The dimensionless parameters *c* and *d* are the same in different areas, they are *c* = −65 and *d* = 8. And the dimensionless parameters *a* and *b* of the neurons in StrD1, StrD2 and other areas are set as *a* = 0.01, *b* = 0.01; *a* = 0.1, *b* = 0.5; *a* = 0.02, *b* = 0.6, respectively.


(1) $$\begin{array}{l} v^{\prime }=0.04v^{2}+5v+140-u+I\\ u^{\prime }=a(bv-u) \end{array}$$

(2) $$ifv\geq 30mV,then\{\begin{array}{ll} v & \leftarrow c\\ u & \leftarrow u+d \end{array}$$

(3) $$\begin{array}{c} I_{ij}=W_{ij}\times O_{ij}\\ O_{ij}=\{\begin{array}{cc} 1 & if\;v_{ij}\geq 30\\ 0 & otherwise \end{array} \end{array}$$

Spike Timing Dependent Plasticity (STDP) is an important learning mechanism in the biological brain, which updates the synaptic weight between presynaptic and postsynaptic neurons according to the time difference between their firing (Bi and Poo, 2001). And the STDP mechanism is widely used in our previous work on brain-inspired cognitive computing modeling (Zeng et al., 2016, 2017, 2020; Zhao et al., 2021b). Within a millisecond time window, if the postsynaptic neurons are fired later than the presynaptic neurons, the synaptic weights between them increase, exhibit the long-term potentiation (LTP) mechanism; if the postsynaptic neurons are fired earlier than the presynaptic neurons, the synaptic weights between them decrease, exhibit the long-term depression (LTD) mechanism. The mathematical description of the STDP mechanism is shown in Equation (4). *A*_+_ and *A*_−_ are the learning rates under the LTP mechanism and the LTD mechanism, respectively. τ_+_ and τ_−_ are time constants for synaptic updates under the LTP mechanism and the LTD mechanism, respectively. To ensure the biologically plausible of the computational model, according to the results of the biological neuron fitting (Bi and Poo, 2001), we set *A*_+_=0.777, *A*_−_=-0.237, τ_+_=16.8, and τ_−_=-33.7 ms. To enable the robot to learn the user's flexible intentions more quickly, the synapse weights are updated according to the ratio based on the current weight, as shown in Equation (5). In the computational model, synaptic plasticity occurs between DLPFC and BG, and the synaptic weight is fixed between other brain areas.


(4) $$\begin{array}{l} \text{Δ}w\text{=}\{\begin{array}{c} A_{+}\times e^{\left(\text{Δ}t/\tau_{+}\right)}\;if\text{ Δ}t<0\\ A_{-}\times e^{\left(\text{Δ}t/\tau_{-}\right)}\;if\text{ Δ}t\geq 0 \end{array}\\ \text{Δ}t=t_{\text{DLPFC}}-t_{\text{BG}} \end{array}$$

(5) $$W{\left(t+1\right)}_{ij}=W{\left(t\right)}_{ij}+W{\left(t\right)}_{ij}\times \text{Δ}w$$

The stimulation transmitted from MOFC to BG and DLPFC exists as follows: the neurons in DLPFC fired first, and the neurons in StrD1 fired later, the Δ*t* is less than 0. And the synaptic weight between DLPFC and BG increased, exhibiting the LTP mechanism. The stimulation transmitted from LOFC to BG and DLPFC exists as follows: the neurons in StrD2 fired first, and the neurons in DLPFC fired later, the Δ*t* is greater than 0. And the synaptic weight between DLPFC and BG decreased, exhibiting the LTD mechanism. Therefore, when the user gives the right feedback, the weight of intention options selected by the model increases, while the weight of other candidate intention options decreases. When the user gives wrong feedback, the weight of intention options selected by the model decreases, while the weights of other candidate intention options are unchanged.

## 3. Results

We deploy the model on the humanoid robot NAO, and verify the effectiveness of the model through Human Intention Prediction Experiment and Trajectory Tracking Experiment.

### 3.1. Human intention prediction experiment

#### 3.1.1. Experimental settings

The Human Intention Prediction Experiment allows the robot to predict human intentions through human gestures (the intention refers to the action that human expects the robot to perform), and to learn new intentions when human intentions change. After 12 gestures and 12 intentions are defined, the user can define the gesture-intention corresponding rules in his mind. The user makes gestures and the robot recognizes the gesture. Then the robot predicts the user's intention and performs the corresponding actions according to the proposed model. The user gives the right or wrong feedback according to whether the robot's action complies with his intentions. The robot can successfully predict the user's intention through multiple interactions. If some of the user's gesture-intention rules change, the robot can continue to learn those changed rules through interaction based on the learned model, and the unchanged rules are not affected, that is, the robot does not need to relearn all the rules.

**Figure 3:**
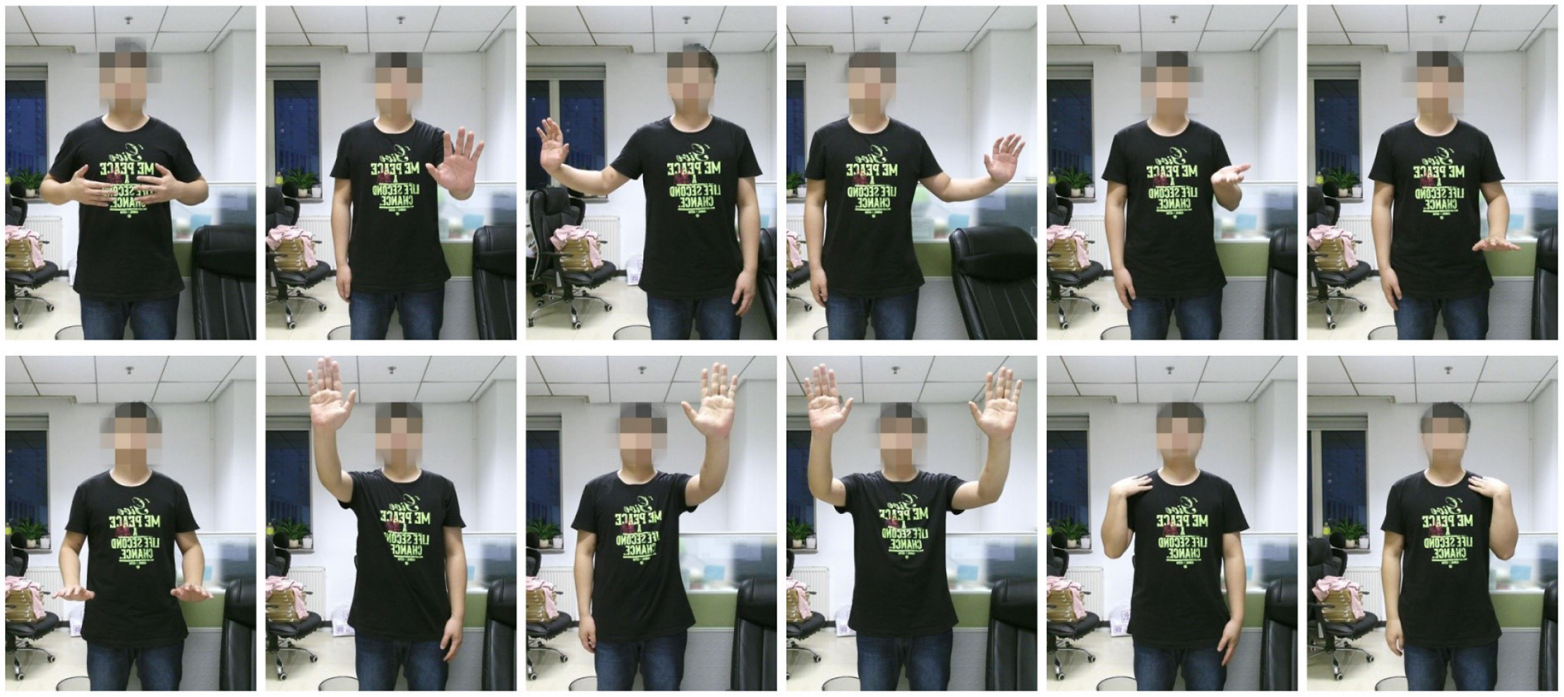
Predefined 12 gestures. From left to right are: Gesture A, Gesture B, Gesture C, Gesture D, Gesture E, Gesture F, Gesture G, Gesture H, Gesture I, Gesture J, Gesture K and Gesture L.

The predefined 12 gestures are shown in Figure 3. Gesture A, both hands close to the body; Gesture B, single hand away from the body; Gesture C, single hand moves to the left; Gesture D, single hand moves to the right; Gesture E, single hand moves up; Gesture F, single hand moves down; Gesture G, both hands move down; Gesture H, single hand above the left shoulder; Gesture I, single hand above the right shoulder; Gesture J, both hands above both shoulders; Gesture K, left hand and left shoulder overlap; Gesture L, right hand and right shoulder overlap.

**Figure 4:**
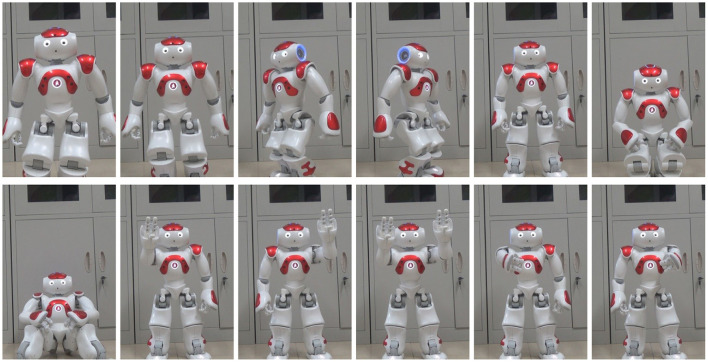
Predefined 12 intentions. From left to right are: Move forward, Move backward, Turn left, Turn right, Stand up, Squat down, Sit down, Clap the left palm, Clap the right palm, Clap both palms, Beat the left shoulder and Beat the right shoulder.

The predefined 12 intentions are shown in Figure 4. The intentions can be roughly divided into three categories: movement intentions (move forward, move backward, turn left, turn right, stand up, squat down and sit down), interaction intentions (clap the left palm, clap the right palm and clap both palms), and service intentions (beat the left shoulder and beat the right shoulder).

**Figure 5:**
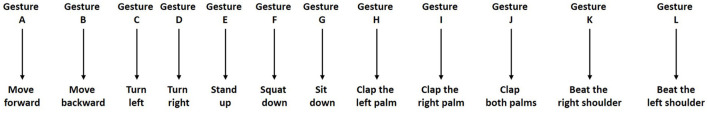
Gesture-intention corresponding rules.

To make the experiment more intuitive, the initially defined gesture-intention correspondence rules are shown in Figure 5.

**Figure 6:**
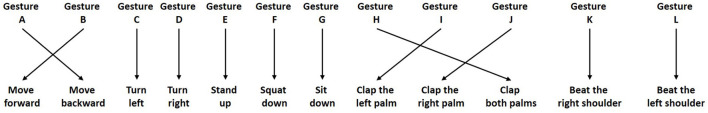
Changed gesture-intention corresponding rules.

After learning the gesture-intention corresponding rules, the user modified the corresponding rules (as shown in Figure 6) to verify the flexibility of the model, and then the robot continued learning through interaction.

#### 3.1.2. Experimental results

Considering that gesture recognition is not the focus of the proposed model, to recognize the user's gestures more simply, we used a Kinect camera for image acquisition. The Kinect camera can capture 25 user's joints and record their three-dimensional space coordinate. We defined 20 neurons to represent the movement direction of the left and right hands (upward movement, downward movement, left movement, right movement, close to the body and away from the body), as well as the position information of the left and right hands compared with the left and right shoulders (overlap with the left shoulder, overlap with the right shoulder, higher than the left shoulder and higher than the right shoulder). These neurons determine whether to fire based on the three-dimensional coordinate information of the joint.

After obtaining the gesture features, we use an unsupervised learning algorithm based on the STDP mechanism for gesture recognition. When a gesture is detected, the correlation coefficient of neurons firing pattern between the detected gesture and the learned gesture is calculated, and the activated target neuron is determined according to the correlation coefficient. If the correlation coefficient is very small, the gesture is determined as a new gesture, and a new target neuron is activated. The synaptic weights update between the new gesture and the new target are based on the STDP mechanism.

The method is an online learning method, and the recognition accuracy increases with the increase of the number of training samples. We define a trial training set that contains 12 types of gestures, each of the gestures is performed once. After the previous trial training ends, the next trial continues learning on the trained model. Test at the end of each trial. The test set consisted of 12 types of gestures performed 30 times each, with a total of 360 samples. When the training of the sixth trial is completed, the gesture recognition accuracy is 98.33%.

The method is an online learning method, and the recognition accuracy increases with the increase of the number of training samples. The training set consists of repeated batch training sets. We define a batch training set that contains 12 types of gestures, each of the gestures is performed once. That is, there are 12 samples in a batch training set, which are different types of gestures. A batch training indicates that the model is trained on the batch training set. The online learning method of the model is realized in the following ways: after the previous batch training ends, the next batch continues learning on the trained model. Test at the end of each batch training. The test set consists of 12 types of gestures, each of which is executed 30 times. The test set includes 360 samples. When the training of the sixth batch is completed (that is, from the initial training, a total of six batches of training were carried out, each batch containing 12 gestures), the gesture recognition accuracy is 98.33%.

**Figure 7:**
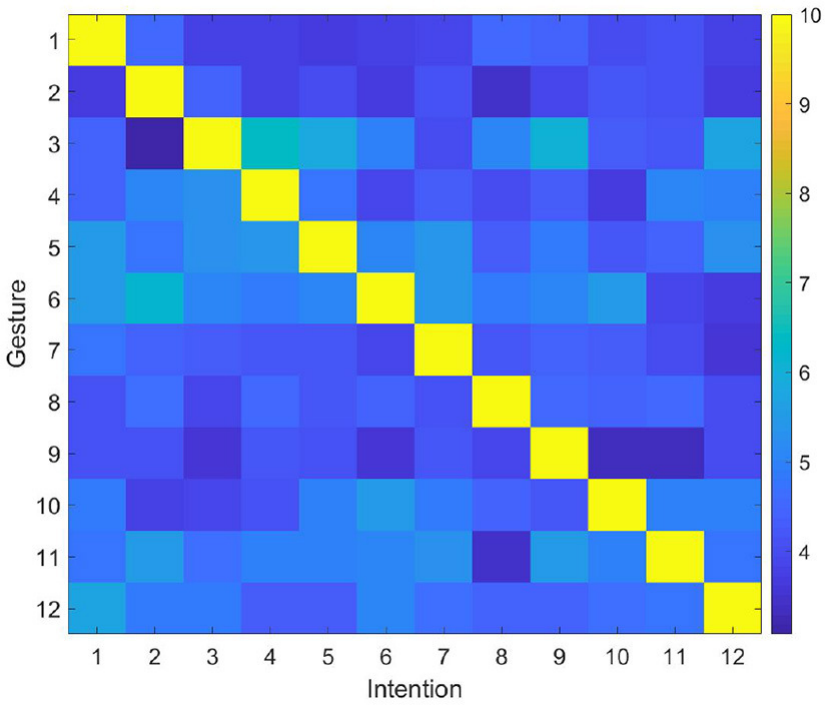
Synaptic weights between DLPFC and BG.

**Figure 8:**
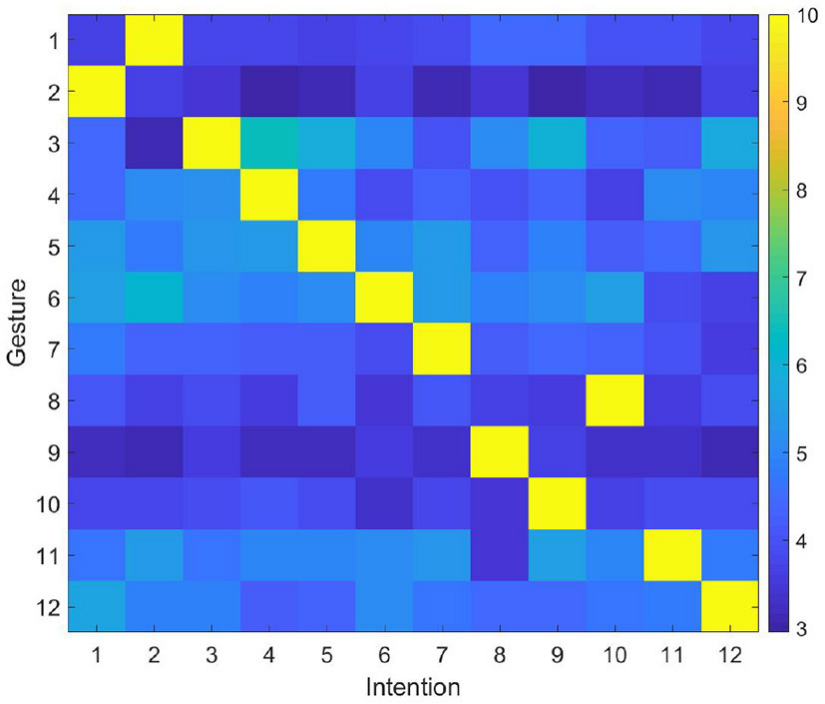
Synaptic weights between DLPFC and BG (Rules changed).

The user makes gestures randomly, and the robot predicts the user's intention and performs the corresponding action through the proposed model. Then, the user gives right or wrong feedback according to the robot's action and the initially defined gesture intention correspondence rules. The experiment is repeated many times until the robot could successfully predict the user's intention. The synaptic weights between DLPFC and BG are shown in Figure 7. In general, the number of interactions required to complete the training ranges from 12 (the robot successfully predicts the intention of the gesture each time) to 78 times (the robot tries all possible intentions until the last time to successfully predict the intention). In most cases, the robot needs 45 interactions to complete the training. Then the user modifies the corresponding rules, and gives feedback according to the modified rule. After modifying the rules, the synaptic weights between DLPFC and BG are shown in Figure 8. Since the synapse weights are updated according to the ratio based on the current weight, when the rules change, the robot can quickly forget the old rules. In general, after two interactions, the robot can forget the old rules and start learning the new ones.

### 3.2. Trajectory tracking experiment

#### 3.2.1. Experimental settings

The Trajectory Tracking Experiment can make the robot learn to walk along the track only through the right and wrong feedback of the remote control.

**Figure 9:**
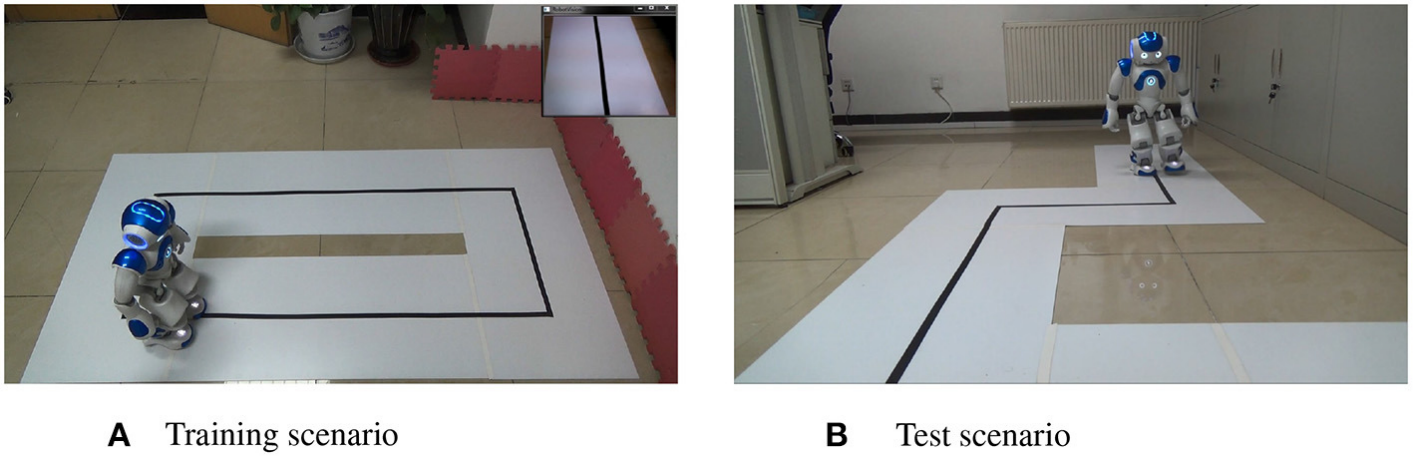
**(A)** Training scenario. **(B)** Test scenario.

**Figure 10:**
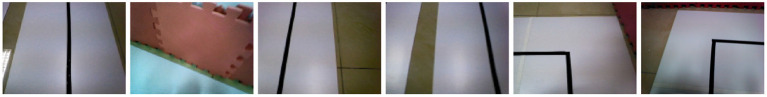
The actions that the user expects the robot to perform according to different images. From left to right are: Move forward (the black line is in the center of the visual field), Move backward (no black line detected), Move left (the black line is on the left side of the visual field), Move right (the black line is on the right side of the visual field), Turn left (the black line turns left) and Turn right (the black line turns right).

The training scenario and test scenario are shown in Figures 9A,B, respectively. In the training scenario, the robot makes behavioral decisions based on the collected image information and the proposed model, such as move forward, move backward, move left, move right, turn left and turn right. Then the user gives right or wrong feedback based on the robot's behavior, and gradually makes the robot learn to walk along the black track. The upper right corner of Figure 9A is the collected image information by the robot. Compared with the training scenario, the test scenario includes turn left and turn right behaviors in a tracking experiment Figure 10.

#### 3.2.2. Experimental results

We detect the trajectory in the image through traditional image processing methods such as image binarization, edge detection, and Hough transform, and classify the trajectory according to its image characteristics. Finally, we use six neurons to implement an abstract representation of the results. The detection method is simple and effective, which can ensure the robot identifies the trajectory with high accuracy.

In the training scenario, the robot completed the training by walking two times along the trajectory clockwise and two times counterclockwise. In the test scenario, the robot can successfully complete the trajectory tracking experiment.

### 3.3. Compared with Q-learning method

First, we test the training times required by the Q-learning method (more details can be found in Supplementary material) and the proposed model under different number of intentions (form 1 to 9). All the intention-action corresponding rules are considered in the test process, and the number of rules corresponding to different intentions is shown in Table 1. The number of rules refers to the number of the intention-action corresponding rules, which is the total number of permutations of intention-action corresponding rules. For the intention-action corresponding rules under the same intention number, the training times required by the Q-learning method are fixed, while the training times required by the proposed method is slightly different according to different rules. In order to better compare the performance of the proposed method, the mode of training times [The Proposed Model (Mode)], minimum training times [The Proposed Model (Min)], and maximum training times [The Proposed Model (Max)] required by the proposed method under different rules are selected for comparison.

**Table 1:** Number of rules under different intention numbers, and the comparison results of Q-learning method and the proposed model.

Number of intentions	1	2	3	4	5	6	7	8	9
Number of rules	1	2	6	24	120	720	5,040	40,320	362,880
Q-learning method	1	3	6	10	15	21	28	36	45
The proposed model (Mode)	1	2.5	4.5	7	10	13.5	17.5	22	27
The proposed model (Min)	1	2	3	4	5	6	7	8	9
The proposed model (Max)	1	3	6	10	15	21	28	36	45

**Figure 11:**
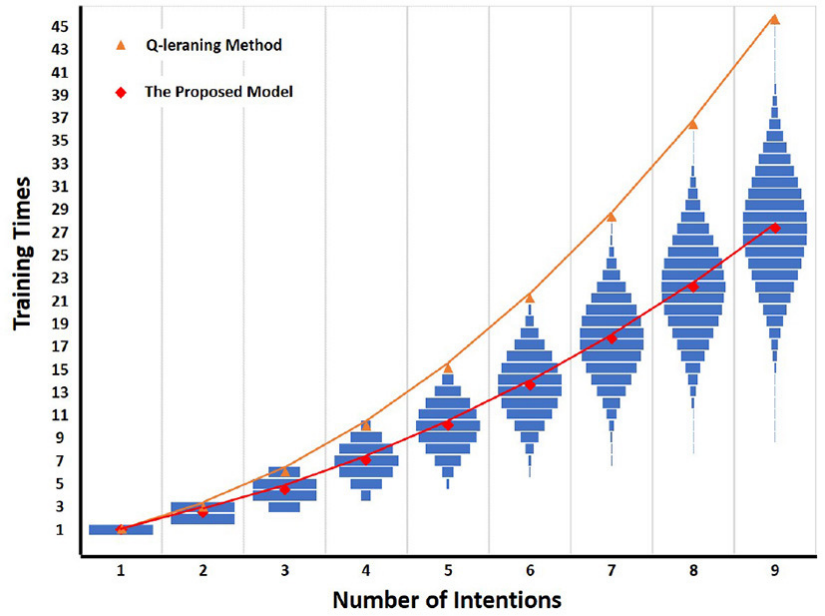
Detailed comparison between Q-learning and the proposed model. The horizontal axis is the number of intentions, and the vertical axis is the number of training times. The blue bar is the training times of the proposed model under different intention-action corresponding rules. The red diamond is the mode of training times required by all intention-action corresponding rules under different intention numbers. If there are multiple modes, the mean value is taken. The orange triangle is the training times of Q-learning method under different intention numbers.

The result of detailed comparison between Q-learning method and the proposed model is shown in Figure 11. From Figure 11 and Table 1, it is easy to see that compared with the Q-learning method, the number of training times required by the proposed model decreases significantly with the increase of the number of intentions. Taking the number of intentions as 6 as an example, the number of all intention-action corresponding rules is 720. The Q-learning method requires 21 training times to complete the training, while the proposed model requires at least 6 times and at most 21 times under different rules. The mean of mode is 13.5 times. In general, the proposed model needs 13.5 times to complete training, which is 7.5 times less than Q learning method.

As can be seen from Figure 11, the training times of the proposed model under different rules are symmetrically distributed, so its mode is equal to the average value. Therefore, when the number of intentions is N, the improvement effect (*Train*_*improve*_) of the proposed model on training times can be calculated by Equation (6). The *Train*_*Q*_ is the training times required by Q-learning method, and the *Train*_*BIIP*_ is the mode of training times required by the proposed model under the given intention numbers.


(6) $$\begin{array}{l} Train_{improve}=Train_{Q}-Train_{BIIP}\\ \;=(1+N)*N/2-(N+(1+N)*N/2)/2\\ \;=(N^{2}-N)/4 \end{array}$$

**Figure 12:**
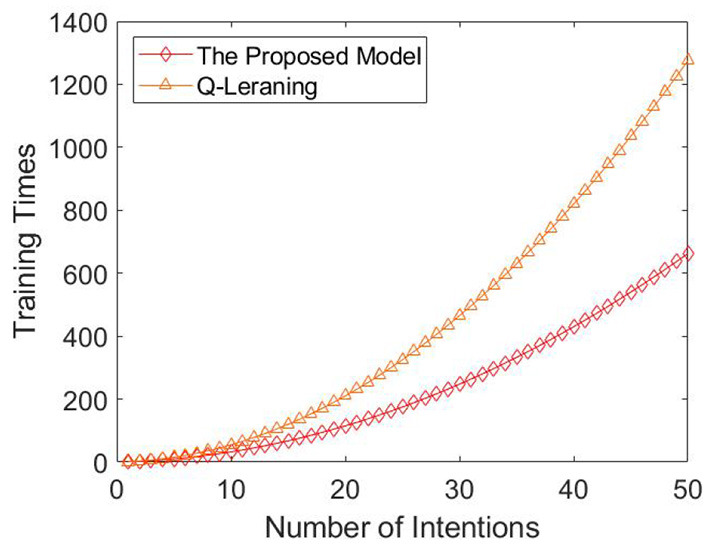
Comparison between Q-learning and the proposed model. The horizontal axis is the number of intentions, and the vertical axis is the number of training times. The red diamond is the mode of training times required by all intention-action corresponding rules under different intention numbers. If there are multiple modes, the mean value is taken. The orange triangle is the training times of Q-learning method under different intention numbers.

Finally, we compared the training times required by the two methods when the number of intentions is 1–50. The experimental result is shown in Figure 12.

## 4. Discussion

Based on the neural mechanism of reinforcement learning, we propose a brain-inspired intention prediction spiking neural network model to enable the robot to perform actions according to the user's intention. With the STDP mechanisms and the simple feedback of right or wrong, the humanoid robot NAO could successfully predict the user's intentions in Human Intention Prediction Experiment and Trajectory Tracking Experiment. Compared with the traditional Q-learning method, the training times required by the proposed model are reduced by (*N*^2^ − *N*)/4, where N is the number of intentions.

Reinforcement learning, supervised learning and unsupervised learning are considered as the three basic machine learning paradigms. It has been successfully applied to different robotic tasks, such as navigation, manipulation, decision-making in human robot interaction. The Q-learning method is a widely used and very effective reinforcement learning method. Compared with the Q-learning method, our model has two characteristics: biologically plausible and requires fewer training times under the same task.

The biologically plausible of the model helps to reveal the neural mechanism of reinforcement learning in the brain from a computational perspective. We ensured the biologically plausible of the model from three aspects: the network structure, the neuron model and the learning mechanism. The network structure refers to the neural mechanism of reinforcement learning, including the relevant brain regions, the functions of these brain regions and the pathways between these brain regions. The neuron model is Izhikevich neuron model which achieves a good balance in biologically plausible and computational efficiency. The learning mechanism uses the most important STDP mechanism in the biological brain, and the results of the biological neuron fitting are used as the parameters of the computational model.

Compared with Q-learning method, the direct reason that our model needs fewer training times is the inhibition of LOFC by MOFC in the process of positive reward processing. The positive reward process indicates that the robot successfully predicted the user's intention. MOFC transmits the information to BG *via* StrD1 and to DLPFC at the same time. This pathway is used to strengthen the synaptic weight between the current visual input (such as *Visual*_1_) and the prediction intention (such as *Intention*_1_) to ensure that the user's intention can be correctly predicted when the same visual input is received in the future. Meanwhile, MOFC inhibits LOFC, then LOFC transmits the information to BG *via* StrD2 and to DLPFC at the same time. This pathway is used to reduce the synaptic weight between the future visual inputs (*Visual*_*others*_) and the currently predicted intentions (*Intention*_1_), avoid new visual inputs to choose the intentions that have been learned (*Intention*_1_), and promote new visual inputs to select other unlearned intentions (*Intention*_*others*_).

## 5. Conclusion

We propose a brain-inspired intention prediction model based on the neural mechanism of reinforcement learning. We deploy the model on the humanoid robot NAO, and verified the effectiveness of the model through Human Intention Prediction Experiment and Trajectory Tracking Experiment. The experimental results show that the robot could successfully predict the user's intentions only through the simple feedback of right or wrong. In this way, the robot can quickly learn new rules without interfering with the learned and unchanged intention rules. The proposed model is simple and effective, which can effectively improve the flexibility and simplicity of human-robot interaction.

In our future work, we will combine our previous work in affective states recognition (Zhao et al., 2020, 2021a) to explore the potential of the proposed model in affective interaction tasks and improve the naturalness and flexibility of human-robot interaction.

## Data availability statement

The python scripts can be downloaded from the GitHub repository of the brain-inspired cognitive intelligence engine at Research Center for Brain-inspired Intelligence, Institute of Automation, Chinese Academy of Sciences: https://github.com/BrainCog-X/Brain-Cog/tree/main/examples/Social_Cognition/Intention_Prediction. The script is based on the brain-inspired cognitive intelligence engine (BrainCog), more details could be found at https://github.com/BrainCog-X/Brain-Cog. Further inquiries should be directed to the corresponding author.

## Author contributions

All authors conceived the initial idea, designed the model, carried out the experiments, and wrote the manuscript.

## Funding

This work is supported by the Strategic Priority Research Program of the Chinese Academy of Sciences (Grant No. XDB32070100), the new generation of artificial intelligence major project of the Ministry of Science and Technology of the People's Republic of China (Grant No. 2020AAA0104305), the Beijing Municipal Commission of Science and Technology (Grant No. Z181100001518006), the Key Research Program of Frontier Sciences, CAS (Grant No. ZDBS-LY-JSC013).

## Conflict of interest

The authors declare that the research was conducted in the absence of any commercial or financial relationships that could be construed as a potential conflict of interest.

## Publisher's note

All claims expressed in this article are solely those of the authors and do not necessarily represent those of their affiliated organizations, or those of the publisher, the editors and the reviewers. Any product that may be evaluated in this article, or claim that may be made by its manufacturer, is not guaranteed or endorsed by the publisher.
