# Supplementary material for: A brain-inspired intention prediction model and its applications to humanoid robot
Source: Front Neurosci. 2022 Oct 21;16:1009237. doi: 10.3389/fnins.2022.1009237 (PMC9633960; doi:10.3389/fnins.2022.1009237)
Supplement: Supplementary file 1 [file Data_Sheet_1.PDF]

# Supplementary Material

## Q-LEARNING METHOD

Reinforcement learning, supervised learning, and unsupervised learning are considered as the three basic machine learning paradigms. It has been successfully applied to different robotic tasks, such as navigation, manipulation, and decision-making in human-robot interaction. The Q-learning method is a widely used and very effective reinforcement learning method. It is a model-free reinforcement learning algorithm. Here, we use the Q-learning method to compare the brain-inspired intention prediction (BIIP) model. The effectiveness of the BIIP model is verified by comparing the training times required by the two models to complete the same task.

The most important thing in the Q-learning method is to learn the Q value matrix (QValue). The Q learning method uses a simple greedy strategy to select the $Action$ corresponding to the current state $j$ (the result of the image category) in the action state matrix (Equation S1). N is the number of intentions.

$$Action = \underset{i}{argmax}(QValue_T[j][i]), \quad j = State, i \in [1, N] \tag{S1}$$

After completing an action selection, it uses speech recognition to obtain feedback, and updates the QValue through Equation S2.

$$QValue_T[j][i] = QValue_{T-1}[j][i] + \alpha * \delta * eTrace_T[j][i] \tag{S2}$$

$QValue_T[j][i]$ is the value to be updated in QValue at the current time T. $\alpha$ is the learning rate, set as 0.85. $\delta$ is the difference generated by feedback, which is calculated by Equation S3.

$$\delta = reward + \gamma * maxQ - QValue_{T-1}[j][i] \quad j = State, i = Action \tag{S3}$$

$\gamma$ is the discount factor, set as 0.9. $maxQ$ is calculated by Equation S4.

$$maxQ = \max_{i}(QValue_{T-1}[j][i]), \quad j = State, i \in [1, N] \tag{S4}$$

The value of the reward is determined by Equation S5. If the feedback is right, the reward is 5. If the feedback is wrong, the reward is -5.

$$reward = \begin{cases} 5 & rightfeedback \\ -5 & wrongfeedback \end{cases} \tag{S5}$$

$eTrace[j][i]$ is the eligibility trace, which is calculated by Equation S6.

$$eTrace_T[j][i] = \begin{cases} 1 & j = State, i = Action \\ \gamma * \lambda * eTrace_{T-1}[j][i] & other \end{cases} \quad \text{(S6)}$$

$\gamma$ is the discount factor, set as 0.9. $\lambda$ is the trace factor, set as 0.8.

The sizes of the $QValue$ and $eTrace$ depend on the number of intentions. If the number of intentions is N, the $QValue$ and $eTrace$ are initialized to the all-zero matrix of NxN.

The input and output of the Q-learning method are the same as that of the BIIP model. The input is the result of the image category, and the output is the behavior of the robot. The former can be regarded as the $State$ in the Q-learning method, while the latter can be regarded as the $Action$ in the Q-learning method.
